# Formation and Change of Chloroplast-Located Plant Metabolites in Response to Light Conditions

**DOI:** 10.3390/ijms19030654

**Published:** 2018-02-26

**Authors:** Yiyong Chen, Bo Zhou, Jianlong Li, Hao Tang, Jinchi Tang, Ziyin Yang

**Affiliations:** 1 Tea Research Institute, Guangdong Academy of Agricultural Sciences & Guangdong Provincial Key Laboratory of Tea Plant Resources Innovation and Utilization, Guangzhou 510640, China; chenyiyong@gdaas.cn (Y.C.); zhoubo@gdaas.cn (B.Z.); Skylong.41@163.com (J.L.); teaishealth@163.com (H.T.); 2 Guangdong Provincial Key Laboratory of Applied Botany & Key Laboratory of South China Agricultural Plant Molecular Analysis and Genetic Improvement, South China Botanical Garden, Chinese Academy of Sciences, Guangzhou 510650, China

**Keywords:** biosynthesis, chloroplast, light intensity, light wavelength, metabolite, photosynthesis

## Abstract

Photosynthesis is the central energy conversion process for plant metabolism and occurs within mature chloroplasts. Chloroplasts are also the site of various metabolic reactions involving amino acids, lipids, starch, and sulfur, as well as where the production of some hormones takes place. Light is one of the most important environmental factors, acting as an essential energy source for plants, but also as an external signal influencing their growth and development. Plants experience large fluctuations in the intensity and spectral quality of light, and many attempts have been made to improve or modify plant metabolites by treating them with different light qualities (artificial lighting) or intensities. In this review, we discuss how changes in light intensity and wavelength affect the formation of chloroplast-located metabolites in plants.

## 1. Introduction

Photosynthesis provides energy source for plant metabolism, and it represents an important photochemical reaction involving the trapping and conversion of energy from sunlight into biological energy in plants. Photosynthesis can be divided into two phases: one phase is the conversion of the photochemical energy from sunlight into biological energy, ATP and NADPH, and the other phase is the conversion of CO_2_ into carbon compounds using the biology energy. Chloroplasts are the organelles in which photosynthesis takes place in plants. Within chloroplasts, chlorophyll absorbs light, and the conversion of the light energy into biological energy is done on the photosynthetic electron transport chain (Calvin cycle). The biological energy is used in assimilatory reactions [1,2,3]. 

Many important metabolic reactions are taken place in chloroplasts, including the biosynthesis of partial amino acids, lipids and fatty acids, vitamins, and isoprenoids, and the reduction of nitrites and sulfates [4,5]. The transportation of the metabolites and messengers between plastids and cytosol are taken place on chloroplast envelopes [6,7]. Chloroplast envelopes contain two layers, the outside envelope which is closed to the cytosolic side, and the inner envelope. Several metabolites, such as lipids and pigments, are biosynthesized in the inner envelope membrane [6,8,9], and chloroplast genome transcription and DNA replication are also implicated within the inner envelope [10]. The chloroplast stroma contains many enzymes, and the carbon assimilation and many biosynthetic processes are located on it. It also contains transcriptional and translational machinery.

Light is one of the key environmental factors that influence the growth and development of the plants. Light energy is captured by photoreceptors in plants, and they are specialized pigment–protein complexes. They drive photosynthetic processes and respond to changes in light conditions (quality and quantity) through the responses of developmental and physiological known as photomorphogenesis [11,12]. Light also plays an important role in the accumulation of plant metabolite and the changes of morphological structure [13]. The biosynthesis of carbohydrates and some C-based defense compounds, for instance, terpenoids and phenols are affected by photosynthetic rates [14]. N-containing plant secondary metabolites typically increase with decreasing light intensities [15]. However, in shade-tolerant plants, for example *Tabernaemontana pachysiphon*, N-based secondary metabolites were observed to accumulate extensively, but in some plant species demanding high light such as *Rauvolfia vomitoria* did not accumulate remarkably when photosynthesis decreased under low light intensities [16,17]. 

Light-emitting diode (LED) technology as a form of supplementation light source has made many advances in the regulation of plant metabolism. LEDs allow the control of light quality, quantity, and photoperiod for the regulation of plant metabolism. Until recently, many researches paid attention to the influence of LED light on the metabolites of vegetables and field crops, for instance lettuce [18,19], cucumber [20,21], tomato [22], radish, spinach [23], rice [24], and wheat [25]. Massa et al. [26] discussed the effect of LED light on plant yield, while Mitchell et al. [27] and Bergstrand et al. [28] reviewed the application status of LEDs and described the latest developments in greenhouse cultivation, respectively. Moreover, a large number of studies have evaluated the influence of light conditions on plant metabolites, which are mainly formed in chloroplasts [29,30,31,32]. However, few reviews have discussed the influence of light wavelength and intensity on metabolite accumulation in chloroplasts. Therefore, in the present article, we review the formation of chloroplast-located metabolites, and discuss how changes in light wavelength and intensity can affect their formation.

## 2. Chloroplast-Located Metabolites

### 2.1. Carbohydrates

**Figure 1:**
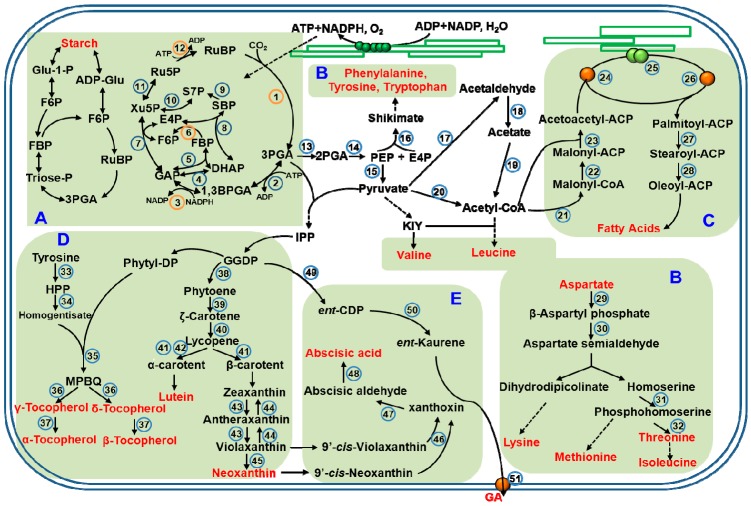
Schematic representation of biosynthetic pathways for the main metabolites in chloroplasts. (This figure is summarized based on review by Gontero et al., 2014 [39]; Joyard et al., 2010 [66]; Lancien et al., 2007 [53]; Kelly et al., 1976 [43]). Different regions represent different metabolite pathways, (**A**) Carbohydrates; (**B**) Amino acids; (**C**) Fatty acids; (**D**) Vitamins; (**E**) Hormones. The enzymes are indicated by number surrounded by a full circle, and orange circle represent sensitive to light-regulation (1, 3, 6, 9 and 12). All the enzymes of these overlapping metabolic pathways are listed in the Table 1. Abbreviations: 2PGA, 2-phosphoglycerate; 3PGA, 3-phosphoglycerate; ACP, acyl-carrier protein; ADP, adenosine diphosphate; ADP-Glu, adenosine diphosphate glucose; ATP, adenosine triphosphate; BPGA: 1,3-bisphosphoglycerate; DHAP: dihydroxyacetone phosphate; E4P: erythrose-4-phosphate; ent-CDP, ent-copalyl diphosphate; F6P, fructose-6-phosphate; FBP: fructose-1,6-bisphosphate; GA, gibberellin; GAP: glyceraldehyde-3-phosphate; GGDP, geranylgeranyl diphosphate; Glu-1-P, glucose-1-phosphate; HPP, p-hydroxyphenylpyruvate; IPP, isopentenylpyrophosphate; KIY, α-ketoisovalerate; NADH, nicotinamide adenine dinucleotide; MPBQ, methyl-6-phytyl-1,4-benzoquinone; NADPH, nicotinamide adenine dinucleotide phosphate; PEP, phosphoenolpyruvate; phytyl-DP, phytyl-diphosphate; Ru5P: ribulose-5-phosphate; RuBP: ribulose-1,5-bisphosphate; S7P: sedoheptulose-7-phosphate; SBP: sedoheptulose-1,7-bisphosphate; Triose-P, triose-phosphate; Xu5P: xylulose-5-phosphate.

Chloroplast is the organelle for photosynthetic carbon assimilation in plant cells. Carbohydrates assimilate in chloroplasts during the day, with some stored as starch, or degraded to supply sugars for growth at night. ATP and NADPH are produced in the photobiochemical process of photosynthesis, and are ultimately consumed in the assembly of CO_2_ into carbon compounds. Once CO_2_ is fixed by ribulose-1,5-bisphosphate carboxylase/oxygenase (Rubisco, EC 4.1.1.39), the product, 3-phosphoglyceric acid (3PGA), is catalyzed by a few reductive and regenerative enzymes and recycled the in the Calvin cycle (Figure 1). Some of reactions in it can be regulated by light, including the reductive activation catalyzed by the key enzymes such as sedoheptulose-1,7-bisphosphatase (FBPase), phosphoribulokinase (PRK), and glyceraldehyde 3-phosphate dehydrogenase (GAPDH) in the ferredoxin/thioredoxin system [33], and protein–protein interactions involving the reversible dissociation of GAPDH and PRK by CP12 (“chloroplast protein of 12 kDa”) [34,35,36,37,38]. The enzymes that have been shown to be sensitive to light regulation are indicated in Figure 1A [39]. 

Starch is synthesized from intermediates of the Calvin cycle. When the rate of photosynthesis exceeds that of sucrose biosynthesis, up to 50% of recently assimilated CO_2_ is stored within the chloroplast as transitory starch. Sachs confirmed that under the condition of light and chlorophyll presence, starch within chloroplasts is directly derived from carbon fixation [40]. The rise in pH caused by light illumination in chloroplast stroma is advantageous for the synthesis of starch [41]. At high pH values, 3PGA is activated by pyrophosphorylase [42], while FBPase activity is enhanced which influences the proportion of carbon flowing through fructose-6-phosphate into starch [43]. In the following periods of darkness, transitory starch within chloroplasts is remobilized, and is used as a continuous carbon source for the development of sink organs and energy metabolism of leaves.

As well as carrying out respiration in darkness, photorespiration is also exhibited in photosynthetic tissues, the light-dependent release of CO_2_, which is suggested to be tightly associated with the metabolism of glycolate, a 2C acid. Glycolate formation is a complex and irreversible process that cannot be uncoupled from photosynthetic carbon metabolism in intact systems. In chloroplasts, glyoxylate is reduced by NADPH-dependent glyoxylate reductase to form glycolate [44,45,46]. Light mainly influences glycolate formation by providing suitable substrates, and glycolate synthesis follows the light saturation curve for photosynthesis [47,48].

### 2.2. Amino Acids

The inorganic nitrogen is assimilated in plants and used to synthesize the 20 amino acids required for the biosynthesis of normal protein. Amino acid synthesis is closely associated with carbohydrate metabolism. Substrates for amino acid synthesis are mainly the intermediates of glycolysis, the citric acid cycle, and the pentose phosphate pathway. For example, aromatic amino acids such as phenylalanine, tyrosine, tryptophan, and histidine are synthesized from erythrose-4-phosphate and phosphoribosylpyrophosphate, which derive from the pentose phosphate pathway (Figure 1B). Phosphoenolpyruvate, one of the other substrates for aromatic amino acid synthesis, comes from glycolysis, while glycine, cysteine, and serine are synthesized from 3-phosphoglycerate, another intermediate of glycolysis. Aromatic amino acids are synthesized within chloroplasts, where ATP and the reductants needed for amino acid biosynthesis are derived [49], together with the essential amino acids [50]. The major metabolic reactions for the essential amino acids biosynthesis and the enzymes that catalyze the reactions are located within chloroplasts [51,52,53]. Oxaloacetate, one of the intermediates of citric acid cycle, is the substrate for synthesis of the aspartic acid derived amino acids asparagine, lysine, threonine, methionine, and isoleucine, while 2-oxo-glutarate is the initial substrate for the biosynthesis of glutamic acid, glutamine, proline, and arginine. Essential amino acids are synthesized from aromatic and aspartic acid derived amino acid pathways, which are the two most-studied pathways. The most part of the pathway for arginine synthesis is localized within the chloroplast, while some enzymes in the pathway are located in the cytosol [54]. Pyruvate is one of the substrates for the biosynthesis of branched-chain amino acids valine and leucine, as well as alanine. The biosynthesis of glutamine and asparagine, two amide amino acids, are tightly regulated by light. For instance, the expression of glutamine synthetase, one of the important enzymes for glutamine, is induced by light, while the asparagine synthetase genes are repressed by light [55].

### 2.3. Lipids

Chloroplasts contain a complex photosynthetic membrane system that dominates the total membrane content of leaves [56] and is the main location for lipid synthesis and assembly [57]. In thylakoids, the chloroplast-specific glycoglycerolipids monogalactosyldiacylglycerol, digalactosyldiacylglycerol, sulfoquinovosyldiacylglycerol, and phosphatidylglycerol construct a special hydrophobic matrix for pigment–protein complexes which are essential for photosynthesis. Some of the fatty acids synthesized in chloroplasts are directly assembled into thylakoid lipids, while others are exported to the endoplasmic reticulum (ER). Chloroplast lipid precursors originate from different locations, and are primarily assembled at the chloroplast envelope membranes [58]. All of the membrane lipids and storage lipids in plant, included fatty acids that derive from plastid-synthesized palmitic acid (16:0) and oleic acid (18:1) are biosynthesized in the chloroplasts stroma (Figure 1C) [59,60]. Once formed, one part of the fatty acids are directly synthesized into glycerolipids (galactolipids, sulfolipid, and phosphatidylglycerol) within the envelopes, while the other parts are exported across the envelopes to the ER in which they are constructed into phospholipids, especially phosphatidylcholine [61,62]. 

Light has been reported to greatly affect chloroplast acyl lipid metabolism. For example, the biosynthesis of the fatty acids is a light-dependent reaction because it needed the reducing power and ATP generated by photosynthesis [63]. Labeled carbon experiments suggested that the most possibility precursor for fatty acid biosynthesis in chloroplasts is pyruvate [64,65]. Acetyl-CoA carboxylase catalyzes the carboxylation of acetyl-CoA to malonyl-CoA in an ATP-dependent manner. C2 units derived from malonyl-CoA then build up 16C and 18C fatty acids. Because malonyl-CoA only functions in fatty acid biosynthesis within plastids, acetyl-CoA carboxylase is considered to be a committed enzyme in the reaction [66]. The extension of the fatty acid chain is catalyzed by a set of essential decentralized enzymes including fatty acid synthase, and all catalytic activities of the enzymes are required for acyl carrier protein. Figure 1C summarizes the formation of 16C and 18C fatty acids in chloroplasts. 

### 2.4. Vitamins

Some plant vitamins are biosynthesized in a restricted compartment. For instance, carotenoids such as pro-vitamin A, vitamins E and K1, and water-soluble riboflavin are biosynthesized in the plastids [67,68,69]. Other vitamins, such as phylloquinone and riboflavin, are biosynthesized not uniformly distributed within plant tissue [70,71]. Carbohydrate metabolites are the main upstream precursors for most vitamins. The most abundant groups of lipid-soluble vitamins, carotenoids and tocopherols, are biosynthesized in partly or in whole pathway of isoprenoid biosynthetic pathway in plastid. 

Carotenoids consist of a large isoprenoid family, most members in which are C40 tetraterpenoids, and phytoene is the substrate for them. Carotenoids can be classified as non-oxygenated and oxygenated, with the latter including xanthophylls. In some flowers and fruits, β-carotene in chloroplasts and lycopene in the chromoplasts are two of the most common non-oxygenated carotenoids. In plant photosynthetic tissues, light-harvesting complexes comprise the most abundant xanthophylls, for instance lutein, iolaxanthin and neoxanthin. Carotenoids are involved in a series of physiological process including the assembly of photosystem, light harvesting, photomorphogenesis, photoprotection, and nonphotochemical quenching [72,73,74,75]. Carotenoids also influence the size and function of the light-harvesting antenna. 

The initial step of plant carotenoid synthesis is the conversion of two molecules of geranylgeranyl diphosphate (GGDP) to formation of phytoene, and the reaction is catalyzed by the enzyme phytoene synthase (Figure 1D). GGDP is considered to be a key intermediate in the pathway for carotenoids, tocochromanols, and many other plastidic isoprenoids biosynthesis [76]. Phytoene is produced as a 15-*cis* isomer, which can subsequently convert to all-*trans* isomer derivatives. Lycopene is biosynthesized from the desaturation of phytoene, the reactions are catalyzed by phytoene desaturase and ζ-carotene desaturase, and the reaction also needed the plastid terminal oxidase and plastoquinone [77,78]. Lycopene is subsequently cyclized to synthesize α-carotene and β-carotene, and then undergo a series of oxygenation reactions, xanthophylls are formed, which are typically found in chloroplasts. After lycopene synthesis, the pathway of carotenoid biosynthesis has two main branches. The difference of the two branches is the cyclic end groups. β-Carotene and its derivatives (zeaxanthin, violaxanthin, antheraxanthin, and neoxanthin) are biosynthesized from β, β branch, which has two beta rings, whereas α-carotene and its derivatives come from β, ε branch, which is defined by one beta and one epsilon ring. The formation of α-carotene and lutein require β-cyclase and ε-cyclase enzymes, respectively [79,80].

Tocochromanols play an important role in the constitution of chloroplast membranes, and are only synthesized in photosynthetic organisms. Tocochromanols are a group of four tocopherols and four tocotrienols, which differ in the degree of methylation of the polar moiety. α-, β-, γ-, and δ-tocochromanols differ only in the number and position of methyl substituents on the aromatic ring. Tocopherols are identified by the phytyl-derived side chain of tocochromanols, while tocotrienols are identified by the geranylgeranyl-derived side chain. The polar moiety of tocochromanols derives from tyrosine, and the hydrophobic polyprenyl side chain originates from the isoprenoid pathway. All tocochromanol synthesis is initiated by p-hydroxyphenylpyruvic acid (HPP) dioxygenase which catalyzes the conversion of HPP into homogentisic acid (HGA). Then the pathway diverges at the step where a polyprenyl side chain is attached to HGA. Homogentisic acid phytyltransferase catalyzes the condensation of HGA and phytyl-diphosphate (phytyl-DP) (Figure 1) to form methyl-6-phytyl-1,4-benzoquinone (MPBQ), which is the committed intermediate of all tocopherols. The other diverged pathway involves the condensation of HGA and solanesyl-diphosphate to form 2-methyl-6-solanesyl-benzoquinol, the immediate precursor of plastoquinone-9. In plants, tocochromanols and the enzymes for their synthesis are localized in plastid membranes [81]. It has been reported that most tocopherols increase under high light stress [82].

### 2.5. Hormones

Abscisic acid (ABA) and gibberellin (GA) are two hormones synthesized in chloroplasts to support photosynthesis [83,84]. ABA is important for seed development and dormancy, as well as plays an important role in response when plants are exposed to environmental stresses. The synthesis and function of ABA are closely related to plastids. In plastids, zeaxanthin (ZEA) is the one of the first products of ABA biosynthesis, which derives from a common five-carbon (C5) precursor, isopentenyl. Pyruvate and glyceraldehyde 3-phosphate are two substrates for isopentenyl diphosphate synthesis, and the intermediate, 2-C-methyl-d-erythritol-4-phosphate (MEP) is key production in the pathway, which is so called MEP pathway. The other pathway for isopentenyl diphosphate is the mevalonic acid pathway, which is located in the cytosol [85]. The biosynthesis of plastidic isoprenoids, including carotenoids, originates from the MEP pathway [86,87]. With the catalysis of zeaxanthin epoxidase, ZEA is then transformed into violaxanthin, and antheraxanthin is the intermediate. The pathway from violaxanthin to neoxanthin synthesis has not been elucidated completely. After the generation of xanthoxin, it is transported into the cytosol and oxidized to form ABA aldehyde, and finally synthesize ABA [88]. Photo-induced ABA signaling can regulate the expression of several thousand nuclear genes [89]. 

GAs are another important hormones, which are essential for multiple processes in the life cycle of higher plants, and it controls plant growth and development [90]. The substrate for GAs biosynthesis is the geranylgeranyl diphosphate (GGDP), which is also an intermediate of carotenoid synthesis, and it is a common C20 precursor for diterpenoids. Terpene synthases (TPSs), cytochrome P450 monooxygenases (P450s), and 2-oxoglutarate-dependent dioxygenases are three different classes of important enzymes, which are required for the transform of GGDP into GAs. *ent*-Kaurene synthase and *ent*-copalyl diphosphate synthase are two TPSs, located in the plastids, and they are key enzymes in the formation of tetracyclic hydrocarbon intermediate *ent*-kaurene [91,92,93]. *ent*-Kaurenoic acid is produced by the oxidized of the C-19 on the *ent*-kaurene, and the reaction is catalyzed by *ent*-kaurene oxidase (KO). *ent*-Kaurenoic acid is sequentially converted to GA12 by *ent*-kaurenoic acid oxidase (KAO) [94]. Experiments have confirmed that KO is present in the outer envelope of chloroplast, whereas KAO is located in the ER (Figure 1E) [92].

### 2.6. Secondary Metabolites

Primary metabolites, such as amino acids, lipids, nucleotides, and so on usually play essential and evident metabolic roles in all plants. While the secondary metabolites, such as terpenoids, phenolic compounds and alkaloids, may not directly participate in plant growth and development [95]. Phenolic compounds are primarily biosynthesized from intermediate of the shikimic acid pathway, and alkaloids are principally biosynthesized from the branch pathway of amino acids. Isoprene, the prime substrate of terpenoids, is produced in plastids. However, sesquiterpenes, triterpenes, and polyterpenes are synthesized in cytosolic and endoplasmic reticulum. It is considered that terpenoids may be the most structurally varied class of plant secondary metabolites. The 5C units of terpenoids can be synthesized by two pathways, acetate/mevalonate pathway or the glyceraldehyde 3-phosphate/pyruvate pathway. In plastids, isopentenyldiphosphate (IPP), which is the precursor for all terpenoidsis, is synthesized from the pathway of glyceraldehyde 3-phosphate/pyruvate [95]. In this pathway, hydroxyethyl- thiamine pyrophosphate (TPP) is produced by the reaction of pyruvate and TPP, and then condenses with glyceraldehyde 3-phosphate. TPP is released to form 1-deoxy-d-xylulose 5-phosphate. Sunlight is one of the most important environmental factors regulating the accumulation of secondary metabolite [96].

## 3. Light Conditions Affect Chloroplast-Located Metabolites in Plants

### 3.1. Light Wavelength Affects Chloroplast-Located Metabolites

Light is one of the most important factors which influence plant metabolite production [97,98]. Light parameters, such as light wavelength, light fluence rate, and photoperiod, affect photosynthesis in plants [99,100]. Plant physiological changes can be triggered by varying light wavelengths [101], and the accumulation of phytochemical metabolites are positively affected by light intensity [102]; however, the effects of light quality are more complex. For example, anthocyanins can be increased by blue light in tomato [103], and by UV-A induction in grape [104] and lettuce [105]. However, in cranberry fruits, anthocyanin production seems most responsive to red light [106]. Moreover, blue light can increase the levels of carotenoids in coffee [107] and ascorbic acid in lettuce and komatsuna, however it has no effect in spinach [32]. These results show that the phytochemical concentration is increased by optimizing light quality. Many studies have focused on red and blue light for the reason that the spectra of them are most important for driving photosynthesis [32,106]. Red light induces transformations in the phytochrome system [108], and blue light affects the formation of chlorophyll, stomatal opening, and photomorphogenesis [109,110,111]. LEDs can be designed to provide different light qualities based on the requirements of plant growth and development [112]. 

The influence of the light wavelength on plant is not only limited to the development and the growth. Recent researches have confirmed that light qualities affect the levels of pigments and metabolites, for instance carbohydrates, amino acids, carotenoids, anthocyanins, fatty acids, and nitrates (Table 2). The conversion of CO_2_ to carbohydrates involves a sequence of light-independent and enzymatic catalytic reactions, but the biosynthesis and accumulation of carbohydrates and proteins are significantly influenced by light qualities. Two major driving photosynthetic biosynthesis lights, red light and blue light are effective at increasing soluble sugar and protein. Many researchers have demonstrated that the soluble sugar content in the seedlings of cucumber, tomato, and radish can be increased by red LED light [113]. In pea seedlings, the levels of soluble sugar can also be markedly increased by red light, whereas the biosynthesis of soluble proteins was significantly restricted [114]. Compared with other types of LED light, blue LED light led to the higher soluble sugar accumulation in tomato seedlings, whereas the level of soluble proteins was highest under a mixture light of red and blue LED [115]. Blue light has also been shown to facilitate protein biosynthesis, and can prevent protein degeneration [116]. During plant growth, blue light exposure is qualitatively required for normal photosynthesis [29].

Plant pigments, chlorophyll and carotenoid, are function in plant light harvesting and photoprotection. Light wavelengths have different effects on these two pigments because their maximum absorptions differ. In the visible light spectrum, the maximum absorption of chlorophyll a and b in the red region is 663 and 642 nm, respectively, and 430 and 453 nm, respectively, in the blue regions. Lutein and β-carotene, which are two carotenoid pigments, have their highest absorption in the blue region at 448 and 454 nm, respectively [117]. It has been reported that total chlorophyll and carotenoid levels were accumulate in white or blue light, but were lower after exposure to red light alone. Compared with white or monochromatic blue light, a mixture of blue LEDs and red LEDs induced higher accumulation of carotenoid levels, whereas carotenoid levels decreased in red light alone. Blue light also increases the accumulation of β-carotene and violaxanthin carotenoid levels [98]. In blue light-activated stomatal operations, zeaxanthin is an important photoreceptor that modulates blue light-dependent responses in plants [118,119].

Light quality also affects the fatty acid profile. Red light promotes the lipid content, and fatty acids can be induced by narrow bandwidth blue light [120]. Compared with other light wavelength treatments of algae, green light significantly increased the concentration of hexadecatrienoic acid (16:3) and resulted in the highest content of α-linolenic acid (18:3), while the concentrations of stearic acid (18:0), oleic acid (18:1), and linolenic acid (18:2) were significantly decreased [121]. In plants and green algae such as *Chlorella vulgaris*, hexadecatrienoic acid (16:3) and α-linolenic acid (18:3) are present in high concentrations in the thylakoid membranes of chloroplasts [121,122]. Because the absorbance of light at a green wavelength is low, the increased proportion of fatty acids together with increases in the number of chloroplasts and/or thylakoid structure rearrangements could compensate for it.

Differences in light quality produce relatively large changes in amino acid levels. Blue light has been shown to enhance the amino acid content, with maize grown under blue light found to have substantially higher amino acid content than that grown under red light or in the dark [120]. Moreover, amino acid production was induced by narrow bandwidth blue light in rice seedling leaves exposed to different LED sources for seven days [123]. UV-B is another light source that regulates the amino acid content. In *Arabidopsis*, UV-B treatment moderately increased the glutamate and γ-amino butyrate content, and dramatically increased that of glutamine and histidine; while the content of these amino acids was unchanged under continuous light conditions. Oxaloacetate- and pyruvate-derived amino acids were also discovered to be changed under the UV-B stress. The lysine content was moderately increased after extending the light period, but dramatically increased following UV-B stress [124]. Other amino acids, such as asparagine, aspartate, and threonine show similar phenomena [124].

Plant hormones such as GA, auxin, cytokinin, brassinolide, and ethylene play an import role in light-regulated development [125]. ABA synthesized from carotenoids is active throughout the development of plant and the responses for stress, and GAs are important in light-regulated seedling development [126]. Light-dependent processes, such as seed germination, photomorphogenesis during de-etiolation, and the photoperiod regulation of stem elongation and flowering are regulated at least in part by GA concentrations. Light, specifically red light, is sensed by phytochromes, and induces seed germination [127]. The GA4 content in seeds can be increased by light treatments which activate phytochromes, elevate the expression of *AtGA3ox1* and *AtGA3ox2*, and suppress that of *AtGA2ox2* [128,129,130]. The expression of *AtGA20ox1* and *AtGA3ox1* genes is downregulated by blue light, which induces *AtGA2ox1* expression. These transcriptional changes correlate with a *cry*-dependent transient decrease in GA4 levels after exposure to blue light [131,132].

### 3.2. Light Intensity Affects Chloroplast-Located Metabolites

The redox state of the photosynthetic electron transport chain is acutely sensitive to fluctuations in light intensity. Light activates a number of biosynthetic enzymes and inhibits glucose-6-phosphate dehydrogenase in carbohydrate degradation. The oxidation of plastoquinol to plastoquinone and the reduction of thioredoxin are favored under low light conditions, while the reverse occurs at higher irradiance [133]. In darkness, this process is fully reversed by oxygen. However, very high light intensity represents an abiotic stress factor for plants, because it may induce secondary destructive processes of photosynthesis. In *Arabidopsis*, the content of most metabolites in glycolysis and the oxidative pentose phosphate pathway were altered when leaves were exposed to high light for six days. This indicated that plants undergo a metabolic shift with high light exposure. Additionally, an increase in the Calvin cycle fixed more carbon, while the activation of photorespiratory pathways was indicated by increased glycine content [134]. The photorespiratory intermediates glycine and glycolate were observed to accumulate in the early phase (5–60 min after transition) of high light stress [135]. Plant photosynthesis can also be damaged by strong UV-B radiation. Under this condition, the activity and content of Rubisco [136] and sedoheptulose 1,7-biphosphatase are both lost [137], while the photosynthetic electron transport chain is inactivated [138], and stomatal closure occurs [139].

The light intensity plays important role in chlorophyll biosynthesis. For instance, Tripathy & Brown found the result that chlorophyll was accumulated in wheat seedlings under red LED light at 100 mmol m^−2^ s^−1^, but not at 500 mmol m^−2^ s^−1^ [140]. In tea leaves, volatile component, for example phenylpropanoids/benzenoids, and some amino acids like l-phenylalanine are increased in darkness induced re-etiolation [141], and the result suggesting the activation of a plastid-located shikimate pathway [142]. We previously showed that most amino acids, including l-phenylalanine, were present at higher levels in dark-treated tealeaves compared with those exposed to light. Our results show that the accumulation of free amino acids in dark-treated tealeaves resulted from the proteolysis of chloroplast proteins rather than the activation of amino acid biosyntheses [143].

The impact of light intensity on allocation to starch is less clear. In *Lolium* and *Sorghum*, high light increased the allocation to starch [144], while starch synthesis raised linearly with the photosynthetic rate at higher irradiance in *Phaseolus vulgaris*, but was negligible at very low irradiance [145]. Britz et al. compared soybean starch across a set of natural light regimes at different times of the year, revealing that starch allocation under natural light regimes was inversely related to photoperiod duration and peak irradiance [146]. Lutein and β-carotene are key components of the light-harvesting complex of leaves, and lutein is important to the plant photoprotection system under high light stress. Niyogi et al. observed photobleachin g and leaf senescence in the absence of lutein under high light intensity [147]. 

## 4. Conclusions

In this review, we summarize the biosynthesis of main chloroplast-located metabolites, carbohydrates, amino acids, lipids, vitamins, and hormones. These metabolites are essential for plant physiological process. Photosynthesis represents one of the most important photochemical reactions for the metabolites biosynthesis in plants, because it’s the central energy conversion process for plant metabolism. Light is the crucial environmental factor to plant photosynthesis. Light conditions, such as light quality and light intensity, can affect the level of plant metabolites. Many attempts have been made to improve or modify plant metabolites by treating them with different light qualities (artificial lighting) or intensities. This review also discusses how plant metabolites affected by changes in light intensity and wavelength.

## Figures and Tables

**Table 1:** List of the enzymes in metabolic pathways that are show in the Figure 1.

No.	Enzyme	No.	Enzyme	No.	Enzyme
1	Ribulose-1,5-bisphosphate carboxylase-oxygenase (RuBisco)	18	Aldehyde dehydrogenase	35	Homogentisate phytyl transferase
2	Phosphoglycerate kinase	19	Acetyl-CoA synthetase	36	Tocopherol cyclase
3	Glyceraldehyde-3-phosphate dehydrogenase	20	Pyruvate dehydrogenase complex	37	γγ-Tocopherol methyltransferase (VTE4)
4	Triose phosphate isomerase	21	Acetyl-CoA Carboxylase	38	Phytoene synthase
5	Aldolase	22	ACP-s-malonyl transferase	39	Phytoene desaturase
6	Fructose-1,6-bisphosphatase	23	β-Ketoacyl-ACP synthase KAS III	40	ζ-Carotene desaturase
7	Transketolase	24	3-Oxoacyl-ACP-reductase	41	Lycopene β-cyclase
8	Aldolase	25	Hydroxyacyl-ACP dehydrase	42	Lycopene ε-cyclase
9	Sedoheptulose-1,7-bisphosphatase	26	3-Enoyl-ACP-reductase	43	Zeaxanthin epoxidase
10	Transketolase	27	3-Oxoacyl-ACP synthase KASII	44	Violaxanthin de-epoxidase
11	Xylulose-5-phosphate epimerase	28	Stearoyl-ACP desaturase	45	Neoxanthin synthase
12	Phosphoribulokinase	29	Aspartate	46	9-*Cis*-epoxycarotenoid dioxygenases (NCED)
13	Phosphoglycerate	30	Aspartate semialdehyde dehydrogenase	47	Alcohol dehydrogenase
14	Enolase	31	Homoserine kinase	48	Abscisic aldehyde oxidase
15	Pyruvate kinase	32	Threonine synthase	49	*ent*-Copalyl diphosphate synthase
16	3-Deoxy-d-arabino-heptulosonate-7-phosphate synthase	33	Tyrosine amino transferase	50	*ent*-Kaurene synthase
17	Pyruvate decarboxylase	34	Hydroxyphenylpyruvate (HPP) dioxygenase	51	*ent*-Kaurene oxidase

**Table 2:** List of chloroplast-located plant metabolites in response to different light conditions. Ref., reference. “+”, represents that the metabolites level increased; “−“, represents that the metabolites level decreased.

Metabolites	Light Conditions	Species	Effects	Ref.
Soluble sugar	Red light	Lettuce; Broccoli; Cabbage; Mustard; Parsley; Maize; Rice	+	[97]
	Red light	Radish	+	[113]
	Red light	Pea seedlings	+	[115]
	Blue light	Spinach; Maize; Cabbage;	+	[97]
	Blue light	Tomato	+	[115]
	High light intensity	Turnip	+	[97]
	Dark	Lettuce; Spinach; Broccoli; Kale; Maize	−	[97]
Starch	High light intensity	Lolium; Sorghum	+	[144]
	High light intensity	*Phaseolus vulgaris*	+	[145]
Glycolate	High light intensity	*Arabidopsis thaliana*	+	[135]
Amino acids	Blue light	Maize	+	[120]
	Narrow bandwidth blue light	Rice	+	[123]
	UV-B	*Arabidopsis thaliana*	+	[124]
	Dark	*Camellia sinensis*	+	[141,143]
Glycine	High light intensity	*Arabidopsis thaliana*	+	[134]
Phenylpropanoids/Benzenoids	Dark	*Camellia sinensis*	+	[141]
Protein	Red light	Pea	−	[114]
	A mixture of red and blue light	Tomato	+	[115]
Lipid	Red light	Maize	+	[120]
Fatty acids	Narrow bandwidth blue light	Maize	+	[120]
Hexadecatrienoic acid	Green light	*Chlorella vulgaris*	+	[122]
α-Linolenic acid	Green light	*Chlorella vulgaris*	+	[122]
Lutein	High light intensity	*Arabidopsis thaliana*	−	[147]
Carotenoids	Red light	Lettuce; Kale; Tomato	+	[97]
	Red light	Lettuce	-	[97]
	Blue light	Coffee	+	[107]
	Blue light	Spinach; Broccoli	+	[97]
	Dark	Kale		[97]
β-Carotene and violaxanthin	Blue light	Broccoli	+	[98]
Zeaxanthin	Blue light	*Lemna trisulca*	+	[119]
GA	Red light	*Arabidopsis thaliana*	+	[128,129,130]
	Blue light	*Arabidopsis thaliana*	−	[131,132]
Anthocyanins	Blue light	Tomato	+	[103]
	UV-A	Grape	+	[104]
	UV-A	Lettuce	+	[105]
	Red light	Cranberry	+	[106]

## Abbreviations

3PGA	3-Phosphoglyceric acid
ABA	Abscisic acid
ER	Endoplasmic reticulum
FBPase	Sedoheptulose-1,7-bisphosphatase
GA	Gibberellin
GAPDH	Glyceraldehyde 3-phosphate dehydrogenase
GGDP	Geranylgeranyl diphosphate
HGA	Homogentisic acid
HPP	p-Hydroxyphenylpyruvic acid
KAO	*ent*-Kaurenoic acid oxidase
KO	*ent*-Kaurene oxidase
LED	Light-emitting diode
MEP	2-C-methyl-d-erythritol-4-phosphate
MPBQ	Methyl-6-phytyl-1,4-benzoquinone
phytyl-DP	Phytyl-diphosphate
PRK	Phosphoribulokinase
Rubisco	Ribulose-1,5-bisphosphate carboxylase/oxygenase
UV-A	Ultraviolet-a radiation
UV-B	Ultraviolet-b radiation
ZEA	Zeaxanthin
